# Microneedle combined with iontophoresis and electroporation for assisted transdermal delivery of *goniothalamus macrophyllus* for enhancement sonophotodynamic activated cancer therapy

**DOI:** 10.1038/s41598-024-58033-7

**Published:** 2024-04-04

**Authors:** Samir Ali Abd El-Kaream, Nabila Gaber Ali Hussein, Sohier Mahmoud El-Kholey, Ahmed Mohammed Abd Elmoez Ibrahim Elhelbawy

**Affiliations:** 1 Applied Medical Chemistry Department, Affiliated Medical Research Institute, Alexandria University, Alexandria, Egypt; 2 Medical Biophysics Department, Affiliated Medical Research Institute, Alexandria University, Alexandria, Egypt

**Keywords:** Biochemistry, Biophysics, Cancer, Medical research, Oncology

## Abstract

The underlying study was carried out aiming at transdermal drug delivery (TDD) of *Goniothalamus macrophyllus* as sono-photo-sensitizer (SPS) using microneedle (MN) arrays with iontophoresis (MN-IP), electroporation (MN-EP) in conjunction with applying photodynamic therapy (PDT), sonodynamic therapy (SDT) and sono-photodynamic therapy (SPDT) as an up-to-date activated cancer treatment modality. Study was conducted on 120 male Swiss Albino mice, inoculated with Ehrlich ascites carcinoma (EAC) divided into 9 groups. We employed three different arrays of MN electrodes were used (parallel, triangular, and circular), EP, IP with different volts (6, 9, 12 V), an infrared laser and an ultrasound (pulsed and continuous wave) as our two energy sources. Results revealed that parallel 6 V TDD@MN@IP@EP can be used as effective delivery system for *G. macrophyllus* from skin directly to target EAC cells. In addition MN@IP@EP@TDD *G. macrophyllus* is a potential SPS for SPDT treatment of EAC. With respect to normal control mice and as opposed to the EAC untreated control mice, MN@EP@IP TDD *G. macrophyllus* in the laser, ultrasound, and combination activated groups showed a significant increase in the antioxidant markers TAC level and the GST, GR, Catalase, and SOD activities, while decrease in lipid peroxidation oxidative stress parameter MDA levels. In addition significantly increased apoptotic genes expressions (p53, caspase (3, 9), Bax, and TNF alpha) and on the other hand decreased anti- apoptotic (Bcl-2) and angiogenic (VEGF) genes expressions. Moreover significantly ameliorate liver and kidney function decreasing ALT, AST, urea and creatinine respectively. Furthermore MN@IP@EP@TDD *G. macrophyllus* combined with SPDT was very effective at reducing the growth of tumors and even causing cell death according to microscopic H&E stain results. This process may be related to a sono- and/or photochemical activation mechanism. According to the findings, MN@IP@EP@TDD *G. macrophyllus* has a lot of potential as a novel, efficient delivery method that in combination with infrared laser and ultrasound activation SPDT demonstrated promising anticancer impact for treating cancer.

## Introduction

Globally, there is a serious public health issue with cancer. The prevalence of cancer is predicted to rise in the coming decades, with 20 million new cases yearly anticipated by 2025. Today, cancer therapies include surgery, chemotherapy, radiotherapy, and immunotherapy. The constraints of each type of treatment, however, present difficult problems for cancer therapy. The overall cancer patient's survival can be increased by diligent research and the creation of new treatment options for malignant tumors. Also cytotoxicity of chemotherapy drugs and traditional cancer treatments results in harmful side effects and an inability to manage the cancer illness. Utilizing medicinal plants is an alternative strategy to counteract the negative effects of synthetic substances. In addition malignant tumors are being treated with novel techniques that are being actively researched and developed, improving patient survival generally. One of cancer alternative therapeutic techniques is sono-photodynamic therapy (SPDT), which uses sensitizer along with ultrasound and photo-irradiation of the tumor to cure disease^1,2^. The use of SPDT to cure various tumors, either alone or in conjunction with other treatment modalities, has gained popularity in recent years. SPDT involves administering a sono-photo-sensitizing (SPS) and then exposing the cancerous area to ultrasound and light with the same absorbance wavelength as the SPS, which causes a number of biological reactions^3–5^. The use of SPSs medicinal compounds derived from plants offers an alternative to the harmful effects of synthetic agents. Many studies have focused on the biochemical effects of phytochemicals derived from plants, but very few have focused on phytochemicals derived from plants. There are about 2,000 species in the 120 genera that make up the *Annonaceae* family. One of the *Annonaceae* genera, *Goniothalamus* has 130–140 species that are found in tropical and subtropical Asia. A medicinal plant known as goniothalamus has compounds that have anticancer effects on a number of tumor cell lines^6,7^.

In recent years, transdermal delivery (TDD) has gained wider acceptance. There have been various attempts over the past few decades to deliver therapeutic chemicals and medications at recommended dosages to the target tissues while minimizing side effects^8^. Transdermal administration of target molecules has received a lot of attention since it is simple to use and requires less intrusive manipulation^9^. This strategy is a novel developed route for delivery that addresses prior drawbacks such low dose delivery, patient discomfort, anxiety, and other adverse effects^10^. Additionally, the potential for medication biodegradation in the gastrointestinal tract and through hepatic activity has been reduced as a result of transdermal distribution. Despite these benefits, cutaneous tissue acquires a significant barrier against numerous external agents with distinct physicochemical features^11^. Normally, the skin has three layers: the outermost epidermis, the middle dermis, and the innermost subcutis. The TDD route's initial target surface for releasing the target substance into the blood circulation is the epidermal layer. Hair follicles, sweat glands, nerve endings, and lymph vessels are all present in the dermis, which is totally vascularized. The local absorption of medicines is facilitated by this tissue layer. The rate-limiting layer for the movement of drug hydrophilic molecules across it is the stratum corneum's (S.C.) intercellular lipid bilayer, which is the topmost layer^12^. Chemical and electrical mechanisms, among other approaches, have been researched to temporarily and locally boost the penetration rate of medicines^13^. By increasing the rate of diffusion across the S.C., chemical agents increase the rate of drug molecules permeability^14^. To transdermal biomolecules and deliver amphiphilic therapeutic compounds to the targeted location with minimal invasiveness, physical approaches such as IP, EP, and MN offer electrical amplification. Both electroporation (EP) and iontophoresis (IP) are used in electrical drug delivery^15^. In order to facilitate the transdermal migration of drugs, physicochemical approaches such as microneedles (MN) is used. IP delivery involves applying small currents to the skin and polarized and neutral molecules moving through it; in the EP technique, tiny pores are created in the skin by applying a 10–1000 voltage for a limited period of time; and MNs, with a little protrusion in their structure, have a cavity for holding medications^10,16,17^.

To our knowledge yet it has not been thoroughly investigated how MN@IP@EP@TDD *G. macrophyllus* works or how it might be used as a SPS for SPDT. Therefore, the primary goal of the current research is to provide a novel study to evaluate the microneedle-iontophoresis-electroporation transdermal delivery of *G. macrophyllus* for activated sono-photodynamic cancer treatment in EAC bearing mice.

## Materials and methods

### Materials

Kits of lipid peroxide (Malondialdehyde; MDA), glutathione-S-transferase and reductase (GST, GSR), catalase (CAT), superoxide dismutase (SOD), total antioxidant capacity (TAC), creatinine, urea, alanine and aspartate aminotransferase (ALT, AST) were obtained from Biodiagnostic (Cairo, Egypt). ABT Total RNA extraction Mini kit (spin column), ABT H-minus synthesis cDNA kit were obtained from (Applied Biotechnology Cat. No: ABT002 and ABT009 respectively) and WizPure™ qPCR Master (SYBR) were obtained from (Wizbiosolutions Inc.)

### Methods

All investigations were carried out in compliance with the guidelines and applicable regulations standards.

### Animals

A total of one hundred and twenty male Albino mice, weighing 20 ± 5 g and having an age range of 60 to 65 days, were bought from the Faculty of Agriculture animal house of the at Alexandria University. Mice were housed in appropriate cages under ambient conditions (25 ± 0.5°C, twelve: twelve sleep-dark/wake-light phase). Mice were given a standard pellet diet and unlimited access to tap water. Before beginning the therapy, mice were given a week to adjust. In a nutshell, mice were divided into 9 groups, each with 10 mice: 1. (−ve Control): normal healthy mice were kept without any treatment. 2. (+ ve Control): mice were received subcutaneous injection (s.c.) of 2 × 10^6^ Ehrlich ascites carcinoma; EAC (in phosphate buffer saline, PBS) once and were kept without treatment. 3a. (MN@EP@IP@TDD *G. macrophyllus*): EAC bearing mice as in group 2 were subjected to EP (5 s) + (parallel) array MN electrodes (6,9,12) volt IP (5 min) with 0.02 ml/mice *G. macrophyllus* (dissolved in PBS) only. 3b. (MN@EP@IP@TDD *G. macrophyllus*): EAC bearing mice as in group 2 were subjected to EP (5 s) + (circular) array MN electrodes (6,9,12) volt IP (5 min) with 0.02 ml/mice *G. macrophyllus* (dissolved in PBS) only 3c. (MN@EP@IP@TDD *G. macrophyllus*): EAC bearing mice as in group 2 were subjected to EP (5 s) + (triangular) array MN electrodes (6,9,12) volt IP (5 min) with 0.02 ml/mice *G. macrophyllus* (dissolved in PBS) only. 4. (Laser group): EAC bearing mice as in group 2 were exposed to Laser 3 min daily for two weeks. 5. (Laser Activated MN@EP@IP@TDD *G. macrophyllus* group): EAC bearing mice as in group 2 were subjected to EP (5 s) + parallel array MN electrodes 6 V IP (5 min) with 0.02 ml/mice *G. macrophyllus*, then were exposed to Laser as group 4. 6. (Ultrasound group): EAC bearing mice as in group 2 were exposed to continuous/pulsed ultrasound 3 min daily for two weeks. 7. (Ultrasound Activated MN@EP@IP@TDD *G. macrophyllus* group): EAC bearing mice as in group 2 were subjected to EP (5 s) + parallel array MN electrodes 6 V IP (5 min) with 0.02 ml/mice *G. macrophyllus*, then were exposed to ultrasound as group 6. 8. (Laser@ultrasound Activated Combined group): EAC bearing mice of this group were exposed to laser light, followed by ultrasound 3 min daily for two weeks. 9. (Laser@ultrasound combined Activated MN@EP@IP@TDD *G. macrophyllus* group): EAC bearing mice as in group 2 were subjected to EP (5 s) + parallel array MN electrodes 6V IP (5 min) with 0.02 ml/mice *G. macrophyllus*, then were exposed to laser followed by ultrasound as group 8.

### Instruments

Three physical approaches were applied to improve transdermal targeted delivery of *G. macrophyllus* as SPS directly from skin to EAC cells for SPDT activated cancer treatment. These methods are MN, EP, and IP. *G. macrophyllus* was applied to an MN electrode. The MN electrodes were then attached to the skin of the selected mice above the tumor with a weight of 1 N/cm2. After 5 s of EP, the IP for 5 min applied. These procedures were carried out for all treatment groups under the previously specified condition of each group.

**Figure 1:**
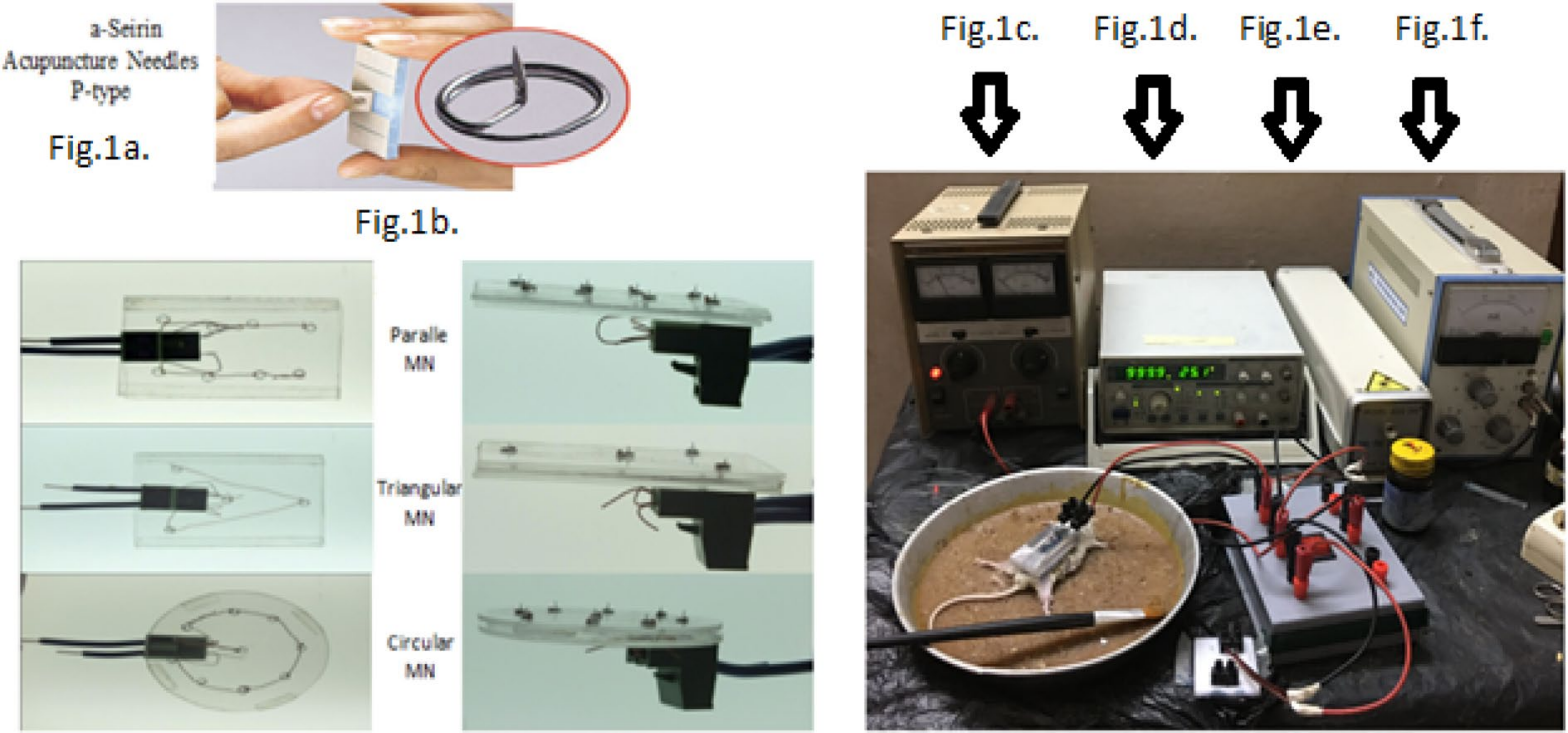
Microneedle, electroporation, iontophoresis assisted transdermal delivery of *G. macrophyllus* [MN@IP@EP@TDD *G. macrophyllus*] (**a**,**b**). (Serine type- parallel, circular and triangular) Microneedle [MN], (**c**). Electroporation [EP], (**d**). Iontophoresis [IP], (**e**). Laser [INRL], (**f**). Ultrasound [US].

**Micro electrode arrays buildup,** in this work, three types of MN arrays were examined. The acupuncture needles (Seirin Acupuncture Needles P-type) (0.22* 1.6 µm) with a 2.8 mm diameter ring handle and the 10 × 10 mm hypoallergenic and waterproof skin tape Fig. 1a were used in their production in the laboratory. For every shape, the MN array's enclosed area measured around 450 mm^2^. (Parallel, circular, and triangle array MN electrodes, Fig. 1b).

**For electroporation,** a pulse generator was used to provide the square wave of EP at 50 V for 5 s. The Power supply is the AC function generator model CA1640 p-02, multi waveforms (sine, square, triangle, etc.) with frequency counter (up to 20 MHz) frequency. The generator was fitted with a power amplifier, capable of giving an AC current to enhance the entrance through the skin Fig. 1c.

**The iontophoresis** desired amount of electric current for experiments was obtained from a DC Power supply type; (DYNASCAN CORP, model 1652), with 240 V maximum DC output, and 0.8 A maximum DC output. The device fitted with a voltmeter and ammeter to adjust both current and voltage taken from it Fig. 1d. This iontophoretic device can produce a direct current up to 0.5 A of 6, 9 and 12 V.

**Laser irradiation**: Diethyl ether was used to anaesthetize the mice before the laser treatment. Around the tumor, the hair was shaved. The mice tumor- was positioned on a board facing up. The probe was placed almost exactly on top of the tumor, which underwent three minutes of laser treatment under the previously specified condition of each group. Mice were kept in the dark after PDT to avoid skin irritation. The laser beam was used to expose the tumor of mice using a laser infrared diode, type LAS 250- Hi-Tech fysiomed, China, with a 600–904 nm wavelength and a peak 50 W output of 7000 Hz frequencies Fig. 1e.

**Ultrasound irradiation**: Diethyl ether was used to anaesthetize the mice before the ultrasonic exposure. Around the tumor, the hair was shaved. The mice tumor- was positioned on a board facing up. The probe was placed almost exactly on top of the tumor, which underwent three minutes of ultrasound treatment under the previously specified condition of each group. An ultrasound (CSl Shanghai, China, Model 822) was used to expose the Ehrlich Tumor. This device employs an electronic tube to generate an alternating current electric oscillation with a frequency of 0.8 MHz and a power output that is transferred to ultrasonic mechanical energy via an ultrasonic transducer. The beam power density of mechanical ultrasonic energy varies from 0.5 to 3W/cm2. This device operates in both pulsed and continuous modes, with 0.5 to 3W/cm^2^ output power range (1000 Hz pulse frequency, duty ratio 1/3, and 0.15 to 1 W/cm^2^ average power density range) Fig. 1f.

Following the completion of the experiment, the mice were anaesthetized by inhaling 5% isofurane. Following dissection, blood samples were collected to acquire both whole blood and sera; one part was centrifuged at 1000 × *g* for 10 min; and the sera separated were stored in − 20 °C until analysis. The remaining collected blood sample in the EDTA-containing vial was transferred to another RNA later solution containing vial and at − 80 °C stored until analysis of identifying relative gene expressions began. Furthermore, EAC tissues were immediately removed, rinsed with cold saline, bored with a needle in a vial, and preserved (in 10% formalin/saline) for histological studies.

### Ethics statement

The Medical Research Institute, Alexandria University (Alexandria, Egypt) Research Ethics Committee (Supplementary Appendix 2; Guide Principles for Biomedical Research Involving Animals, 2011, ALEXU-IACUC; Code No. 01219073022) approved the experimental procedures, animal handling, and sampling in accordance with the Guide for the Care and Use of Laboratory Animals, 8th Edition (National Research Council, 2011). According to the ARRIVE guidelines (https://arriveguidelines.org), this study was reported.

### EAC Tumor growth/inhibition assay

Every day during the therapy period, the tumor's growth was monitored routinely. The following equation was used to compute the tumor volume (in mm^3^) after the tumor's width and length were measured with a slide caliper. TV (mm^3^) = 22/7 × 4/3 × (length/2) × (width/2)^2^. The mice were sacrificed two weeks following the therapy, and the tumors were removed, dissected, and weighed (in grams). Calculations were made to determine the tumor volume growth and inhibition ratio and the tumor mass growth and inhibition ratio.

### Biochemical, molecular, and histological examinations

#### Biochemical analysis of liver and kidney enzymes in serum

Liver (ALT, and AST) enzymes and Kidney (Urea, and Creatinine) were measured according to Burtis et al. (2008) via commercial kits^18^.

#### Determination of the oxidative stress and antioxidant (oxidants/antioxs) markers

Using commercial kits, the levels of oxidants/antioxs markers in the sera was evaluated. Malondialdehyde (MDA) equivalents, which are used to in order to predict lipid peroxidation, were calculated using the Draper and Hadley (1990) method^19^. The SOD activity was evaluated using the Marklund and Marklund (1974) methodology^20^. Based on a modified version of Habing et al.'s (1974) approach, the activities of glutathione-S-transferase and reductase (GST, GR) were assessed^21^. The Catalase activity was measured using the Aebi et al. (1984) technique^22^. The measurement of total antioxidant activity (TAC) followed the methodology proposed by Rice-Evans and Miller, et al. (1994)^23^.

#### Assessment of p53, Caspase (3, 9), Bax, TNF alpha, Bcl-2, and VEGF genes expressions

The real time-polymerase chain reaction (qRT-PCR) was utilized to assess genes expressions. Following the instructions on the ABT Total RNA Mini spin column extraction kit, total RNA was extracted from blood samples. The 260/280 nm absorption ratio, which was typically higher than 1.8, was used to measure purity. The cDNA was created via RT-PCR one-step reaction in accordance with the instructions provided by the ABT H-minus cDNA synthesis kit. WizPure™ qPCR Master (SYBR) with ROX Dye was used to perform qRT-PCR. A reaction mixture (20 µL) containing template C-DNA (5 µL), desired gene primers (0.25 µM) P53-F: CTGTCATCTTCTGTCCCTTC, P53-R: TGGAATCAACCCACAGCTGCA, Caspase3-F: TGTCATCTCGCTCTGGTACG, Caspase3-R: AAATGACCCCTTCATCACCA, Caspase9-F: AGTTCCCGGGTGCTGTCTAT, Caspase9-R: GCCATGGTCTTTCTGCTCAC, TNF-α-F: CACGTCGTAGCAAACCACC, TNF-α-R: TGAGATCCATGCCGTTGGC, Bax-F: CTACAGGGTTTCATCCAG, Bax-R: CCAGTTCATCTCCAATTCG, Bcl2-F: GTGGATGACTGAGTACCT, Bcl2-R: CCAGGAGAAATCAAACAGAG, VEGF-F: AAAAACGAAAGCGCAAGAAA, VEGF-R: TTTCTCCGCTCTGAACAAGG, β-actin-F: AGCCATGTACGTAGCCATCC, β-actin-R: CTCTCAGCTGTGGTGGTGAA and Sybr Green qRT-PCR (10 µL) was used. Thermo Scientific's PikoReal Real-Time PCR apparatus (PR0241401024) used 35 cycles of 95 °C for 10 s, 55 °C for 10 s, and 72 °C for 5 s throughout each run. The 95 °C for 5 min initial denaturation was carried out. All of the gene expressions were compared to the same sample β-actin mRNA levels, and the difference fold was computed by the previously discussed equation 2^−ΔΔCt^^24^.

### Skin and EAC tissue histopathological analyses

After being exposed to various MN arrays, EP, and IP voltages, skin sections from mice were obtained and immediately placed in a deep freezer to be processed by a cryostat microtome and then frozen section stained by eosin dye as counter stain to determine the mean value of pore diameter penetrated through (µm), *G. macrophyllus* transported (µg/cm^2^), absorbed (µg/mg tissue), flux rate (µg/cm^2^/5 min) and penetration depth (µm)^25^. The collected EAC samples were fixed by immersing in a 10% formalin/saline solution, and then sectioned into sections after being imbedded in paraffin. Hematoxylin and eosin (H&E) staining dye was applied to determine the severity of the histological alterations in the EAC tissue. A light microscope was used to inspect and image each slide^26^.

### Statistical analysis

The data was presented as mean ± standard deviation (SD). The results statistical variances were checked employing one-way analysis of variance (ANOVA). A p value ≤ 0.05 was applied to determine statistical significance. The post hoc analysis feature of SPSS 25.0 was used to compare between groups.

## Results

### MN@EP@IP TDD *G. macrophyllus* study results

**Figure 2:**
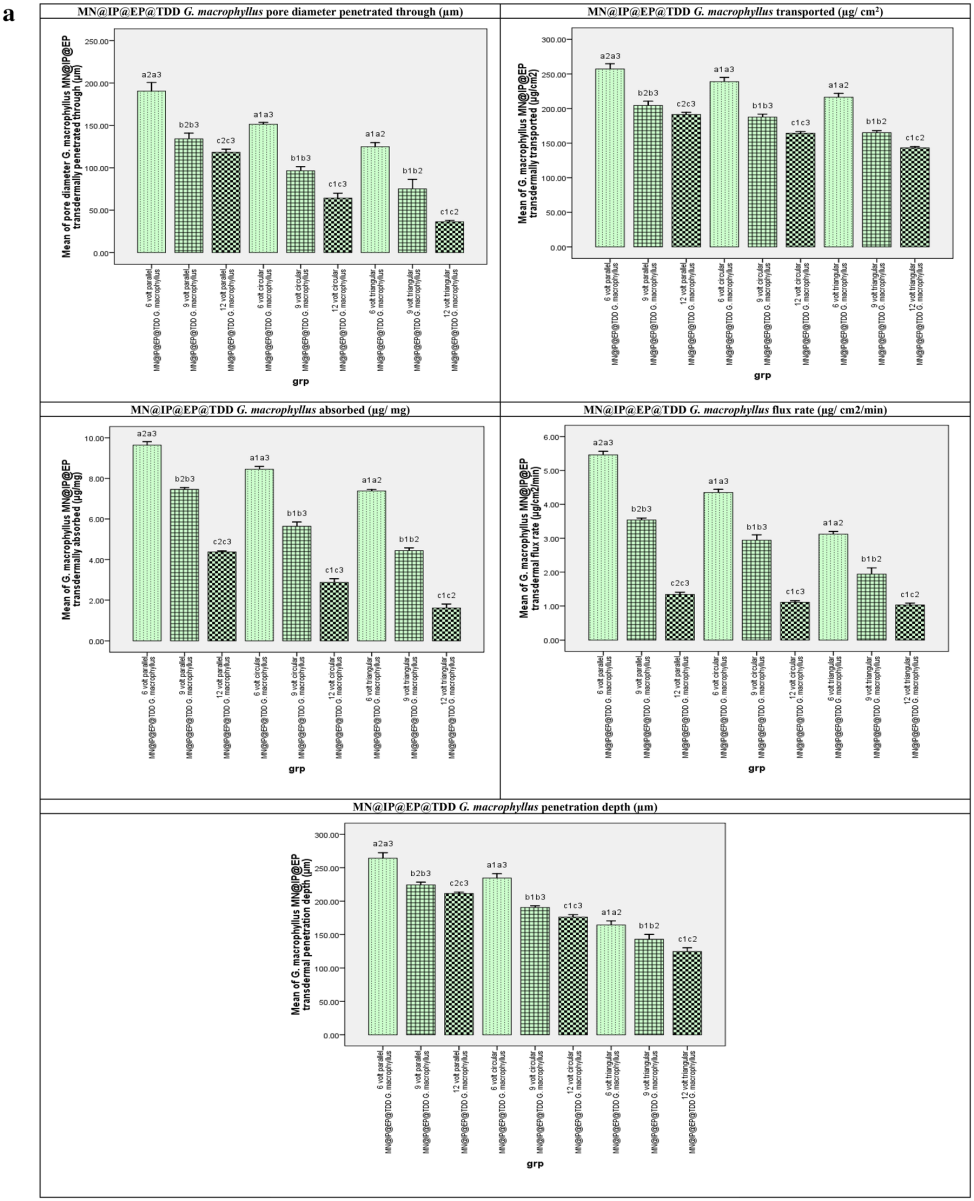

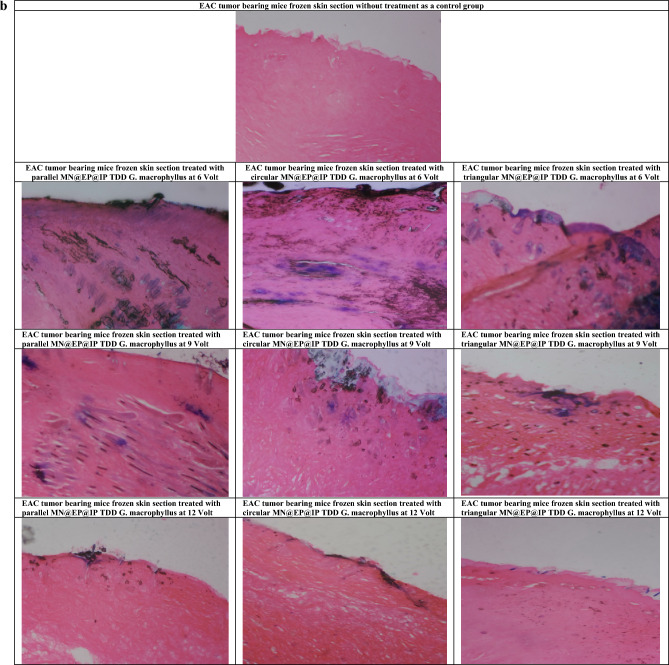
(**a**) MN@IP@EP@TDD *G. macrophyllus* pore diameter penetrated through (µm) F(p) = 237.212(0.001*), transported (µg/cm^2^) F(p) = 1.921E3(0.001*), absorbed (µg/mg tissue) F(p) = 856.956(0.001*), flux rate (µg/cm^2^/5min) F(p) = 272.534(0.001*) and penetration depth (µm) F(p) = 213.125(0.001*) at 5 s electroporation EP for 5s followed by microneedle (parallel, circular and triangular) assisted DC-Iontophoresis (Square wave, 0.5 mA for 5min) at different volts (6, 9 and 12 V for 5min). (**b**) Frozen skin cryo-sections illustrating microneedle assisted electroporation-iontophoresis transdermal delivery of Goniothalmus macrophyllus (MN@EP@IP TDD G. macrophyllus) and penetration depth using different MN arrays and IP volts 2; parallel MN array@EP@IP (6V) TDD, 3; circular MN array@EP@IP (6V) TDD, 4; triangular MN array@EP@IP (6V) TDD, 5; parallel MN array@EP@IP (9V) TDD, 6; circular MN array@EP@IP (9V) TDD, 7; triangular MN array@EP@IP (9V) TDD, 8; parallel MN array@EP@IP (12V) TDD, 9; circular MN array@EP@IP (12V) TDD, 10; triangular MN array@EP@IP (12V) TDD. [1; untreated EAC implanted group skin section without any treatment,].

The effects of MN different array (parallel, circular, triangular)@EP@IP different volts (6, 9, 12 V) on TDD of *G. macrophyllus* are shown in Fig. 2a,b. The cryo-histologic evaluation of the section of *G. macrophyllus* painted EAC bearing mice skin exposed to (parallel, circular, triangular) MN array at (6, 9, 12) volts revealed that applying parallel MN-IP@EP is more effective, followed by circular MN-IP@EP and triangular MN-IP@EP accompanied with 6 more effective than 9 and 12 V respectively. Parallel MN-IP@EP with lower voltage (6 V) is the most efficient for *G. macrophyllus* transdermal delivery application showed deepest penetration of blue green color stain at epidermis and dermis layer.

#### MN@EP@IP TDD *G. macrophyllus* PDT, SDT, SPDT and EAC tumor indices

**Figure 3:**
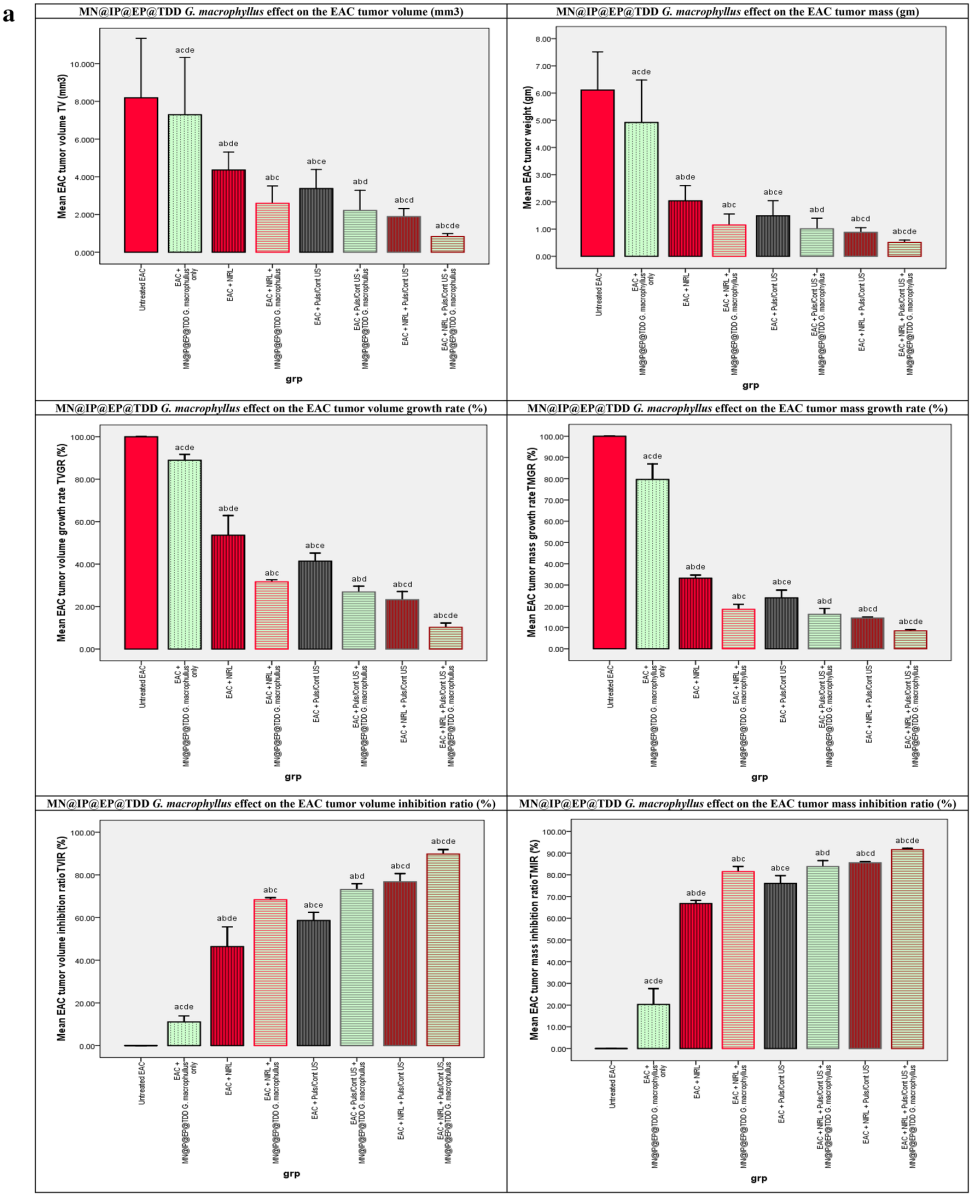

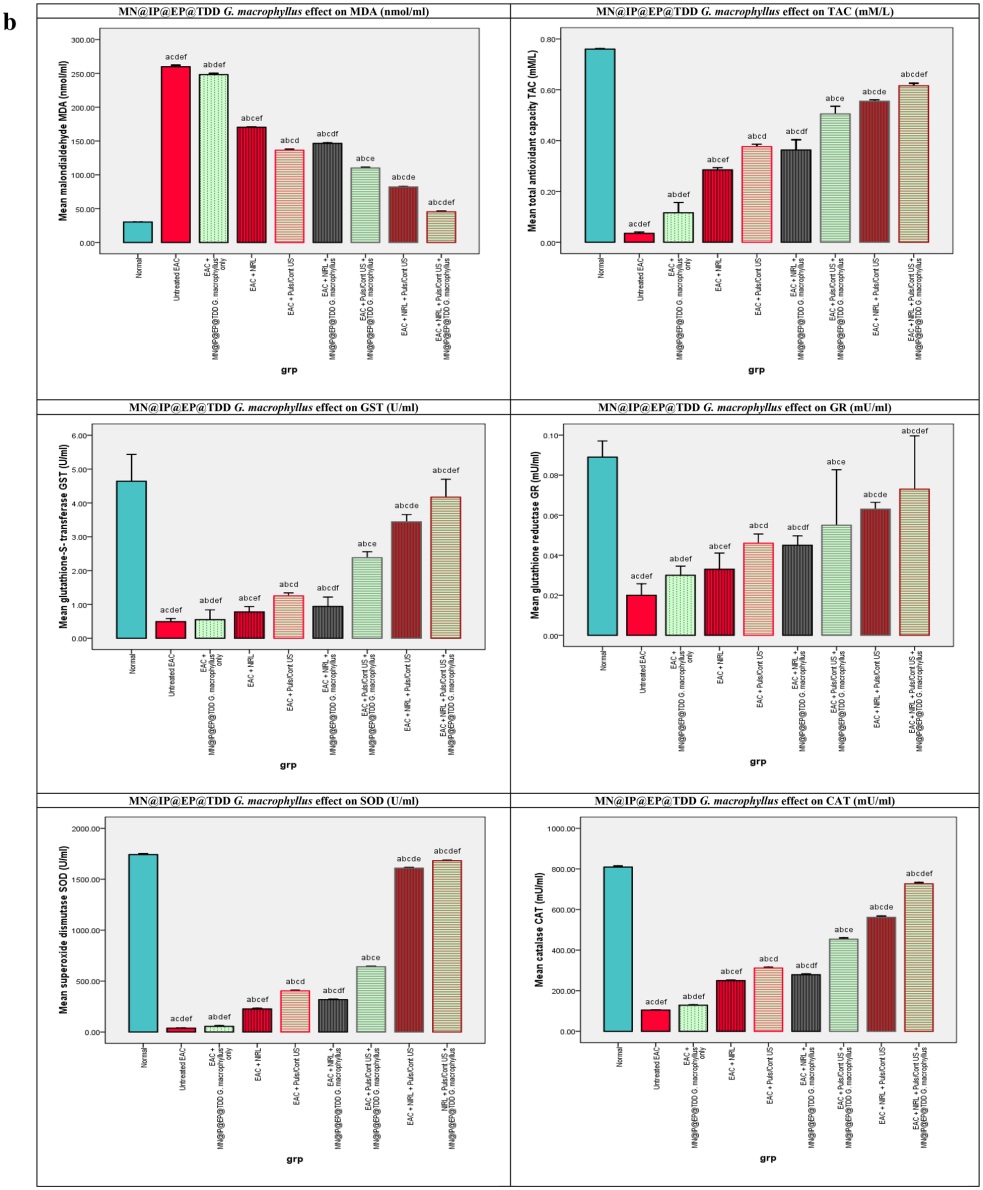

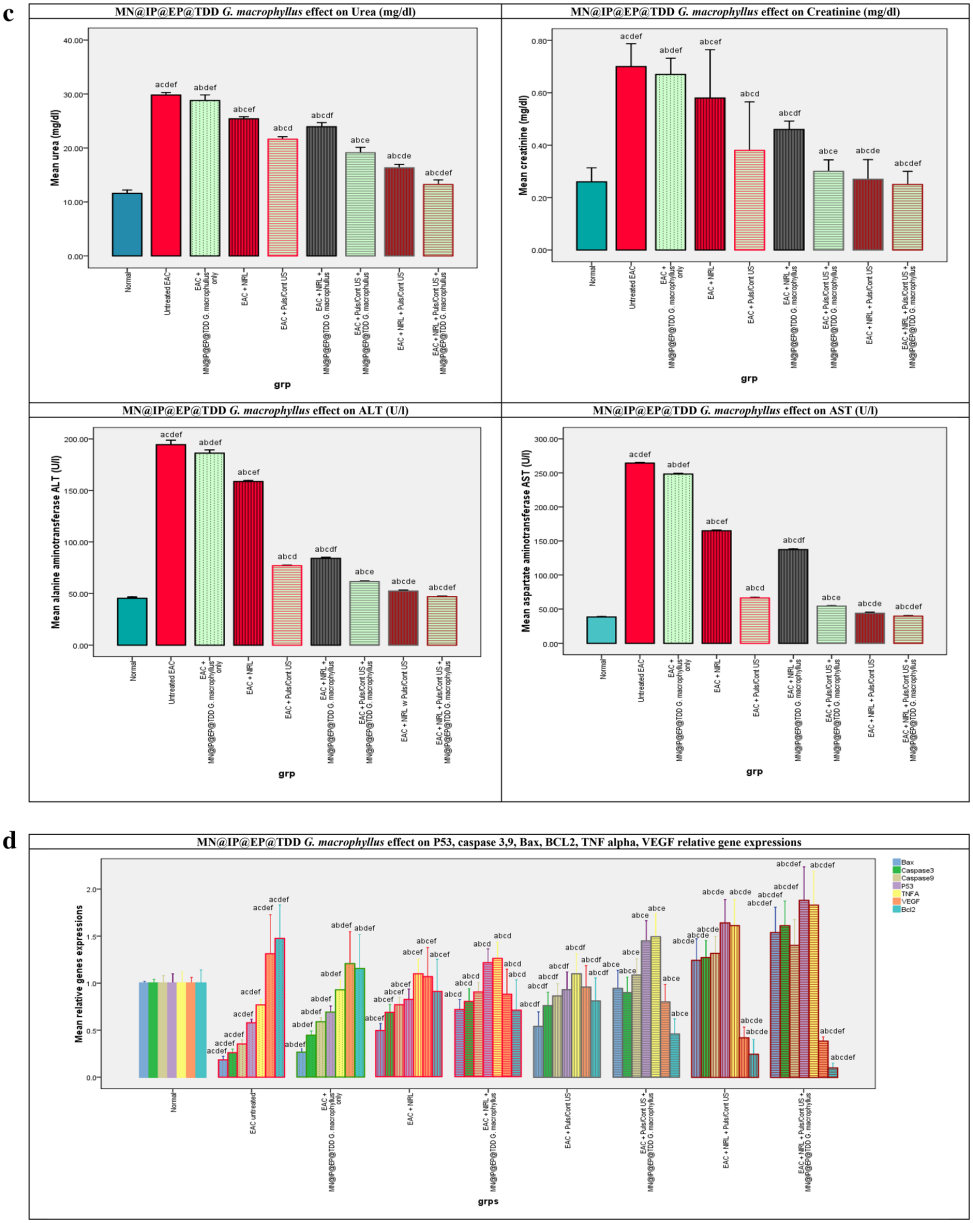
(**a**) The effect of laser, ultrasound continuous/pulsed and Combined modalities on the EAC tumor volume (mm3 ) F(p) = 44.073(0.001*), tumor volume growth rate (%)F(p) = 1.128E3(0.001*), tumor volume inhibition ratio (%)F(p) = 1.128E3(0.001*), tumor mass (gm) F(p) = 25.731(0.001*), tumor mass growth rate (%)F(p) = 449.995(0.001*), tumor mass inhibition ratio (%)F(p) = 449.995(0.001*), of untreated and treated groups. (**b**) The effect of laser, ultrasound continuous/pulsed and Combined modalities on antioxidants activities, capacities and MDA, of untreated and treated groups. F: value for ANOVA test MDA (nmol/ml): 2.149E4 p < 0.001*, TAC (mM/L): 440.982 p < 0.001*, GST (U/ml): 80.472 p < 0.001*, GR (mU/ml): 10.170 p < 0.001*, CAT (mU/ml): 1.695E4 p < 0.001*, SOD (U/ml): 4.113E5 p < 0.001*. (**c**) The effect of laser, ultrasound continuous/pulsed and Combined modalities on renal and hepatic biomarkers, of untreated and treated groups. Urea (mg/dl): 310.998 p < 0.001*, Creatinine (mg/dl): 12.490 p < 0.001*, ALT (U/l): 4.140E3p < 0.001*, AST (U/l): 5.461E4 p < 0.001*. (**d**) p53, Bax, Caspase (9,3), TNF alpha, VEGF, Bcl-2 qRT-PCR relative gene expression of all study groups p53 F(P): F = 67.190 p < 0.001*, Bax F(P): F = 106.888 p < 0.001*, Caspase 9 F(P): F = 66.025 p < 0.001*, Caspase 3 F(P): F = 100.944 p < 0.001*, TNFalpha F(P): F = 33.830 p < 0.001*, VEGF F(P): F = 19.537 p < 0.001*, Bcl-2 F(P): F = 34.116 p < 0.001*.

The effects of MN@EP@IP TDD *G. macrophyllus* PDT, SDT, and SPDT on the EAC tumor indicesis, in all mice study groups are shown in Fig. 3a. Treatment with MN@EP@IP@TDD *G. macrophyllus* only without activation exhibited a merely impact on EAC tumor volume and weight compared to untreated EAC-bearing mice control group. Treatments with laser, ultrasound, or combination in absence of MN@EP@IP@TDD *G. macrophyllus* have slight impact on the tumor indices. Treatment with ultrasound and laser in the presence of MN@EP@IP@TDD *G. macrophyllus*, become more effective. The SPDT combined treatment modality in the presence of MN@EP@IP@TDD *G. macrophyllus* was most potent on tumor cells than applying of ultrasound or laser alone.

#### MN@EP@IP TDD *G. macrophyllus* PDT, SDT, SPDT and EAC oxidative stress

The effects of MN@EP@IP TDD *G. macrophyllus* PDT, SDT, and SPDT on the lipid peroxidation parameter MDA, in all mice study groups are shown in Fig. 3b. Comparing the non-activated group of mice treated with MN@EP@IP TDD *G. macrophyllus* alone to the untreated EAC-bearing mice control group, the MDA exhibited merely significant changes in the sera. In comparison to the normal, healthy control mice, the values of this parameter were considerably higher in the untreated EAC-bearing control mice. Additionally, with regard to the normal control mice, all EAC mice treated with laser, ultrasound, or combination only groups showed a significant increase in MDA concentrations. However, when MN@EP@IP TDD *G. macrophyllus* was administered to the laser, ultrasound and combination activated groups, MDA levels were significantly reduced compared to the EAC control mice but did not achieve the normal control mice.

#### MN@EP@IP TDD *G. macrophyllus* PDT, SDT, SPDT and the antioxidant system

The effects of MN@EP@IP TDD *G. macrophyllus* PDT, SDT, and SPDT on the antioxidant markers (GST, GR, Catalase, SOD, and TAC) in in all mice study groups are shown in Fig. 3b. Comparing the MN@EP@IP TDD *G. macrophyllus* alone without activation to the EAC untreated control group, the TAC level, as well as GST, GR, catalase, and SOD activities, exhibited a merely significant changes. In comparison to the normal control mice, the EAC untreated control mice had significantly reduced GST, GR, Catalase, SOD, and TAC levels. Additionally, with regard to the healthy control mice, all of the EAC mice groups treated with laser, ultrasound, or combination solely groups showed a substantial decrease in the level of TAC and the GST, GR, Catalase, and SOD activities. As opposed to the EAC untreated control mice, MN@EP@IP TDD *G. macrophyllus* in the laser, ultrasound, and combination activated groups showed a significant increase in the TAC level and the GST, GR, Catalase, and SOD activities, but did not reach the normal control group.

#### MN@EP@IP TDD *G. macrophyllus* PDT, SDT, SPDT treatment improved liver functions

The liver function tests in the sera of all studied groups are demonstrated in Fig. 3c. The ALT and AST levels in the sera of mice treated with MN@EP@IP TDD *G. macrophyllus* alone without activation exhibited a merely significant changes from the EAC untreated control group, whereas the levels in the sera of untreated EAC bearing control mice were significantly elevated than those in the normal healthy control mice. Additionally, when compared to the normal control group, all EAC mice treated with laser, ultrasound, or combination alone groups showed a significantly elevated level of ALT and AST. Moreover, when MN@EP@IP TDD *G. macrophyllus* was administered to the laser, ultrasound, and combination activated groups, the level of ALT and AST was significantly decreased with regard to the EAC untreated control mice but still did not achieve the normal control group.

#### MN@EP@IP TDD *G. macrophyllus* PDT, SDT, SPDT treatment improved kidney functions

The kidney function tests in the sera of all studied groups are demonstrated in Fig. 3c. The serum of mice treated with MN@EP@IP TDD *G. macrophyllus* alone without activation exhibited a merely significant changes in the levels of urea or creatinine in comparison to the EAC untreated control mice, whereas the levels of this parameter in untreated EAC bearing control mice were significantly higher with regard to the normal healthy control mice. Additionally, when compared to the normal control group, all EAC mice treated with laser, ultrasound, or combination alone groups demonstrated a considerably significant elevated urea and creatinine level. Moreover, the administration of MN@EP@IP TDD *G. macrophyllus* in the laser, ultrasound, and combination activated groups significantly decreased urea, and creatinine levels compared to the untreated EAC control mice but still did not reach the normal control group.

#### MN@EP@IP TDD *G*. *macrophyllus* PDT, SDT, SPDT; anticancer, antiproliferative and antiangiogenic effects

The impacts of MN@EP@IP TDD *G. macrophyllus* SPDT on the relative gene expression of p53, Bax, Bcl-2, Caspase (3, 9), TNF alpha, and VEGF in all studied groups are presented in Fig. 3d. When mice were given MN@EP@IP TDD *G. macrophyllus* alone without activation, the changes in the gene expression of p53, Bax, Bcl-2, Caspase (3, 9), TNF alpha, and VEGF exhibited a merely significant changes with regard to the EAC untreated control mice, whereas the levels of p53, caspase (3, 9), Bax, and TNF alpha were significantly, lower, while Bcl-2 Bcl-2 and VEGF were significantly higher in untreated EAC bearing mice control group compared to normal, healthy control mice. Additionally, with regard to the healthy control mice, all EAC mice treated with laser, ultrasound, or a combination of the two alone groups showed a significantly decreased level of p53, caspase (3, 9), Bax, and TNF alpha gene expressions and a significantly an elevated level of Bcl-2 and VEGF gene expressions. As opposed to the EAC untreated control group, the administration of MN@EP@IP TDD *G. macrophyllus* to the laser, ultrasound, and combination activated groups significantly increased p53, caspase (3, 9), Bax, and TNF alpha gene expressions and significantly decreased Bcl-2 and VEGF gene expressions.

#### MN@EP@IP TDD *G. macrophyllus* PDT, SDT, SPDT histopathological effect on EAC

**Figure 4:**
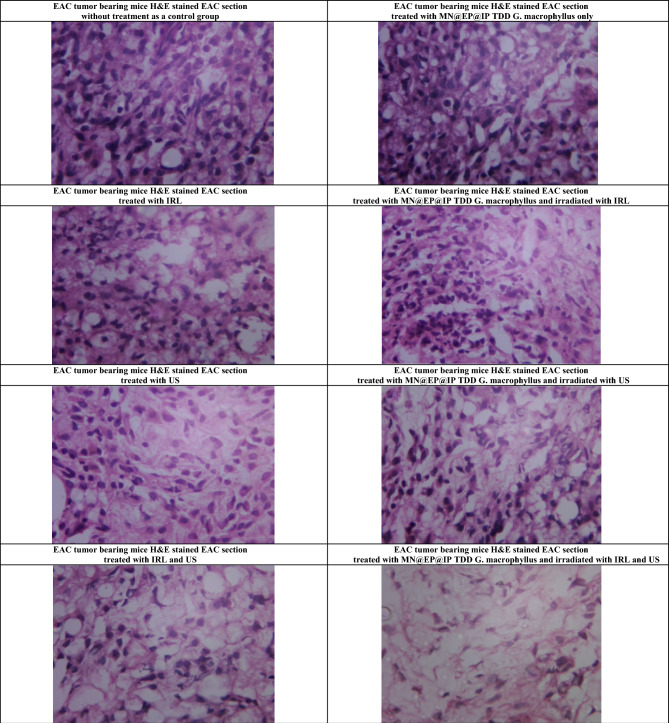
The effect of 3; NIRL only, 4; NIRL in presence of MN@EP@IP TDD *G. macrophyllus*, 5; pulsed/continuous US in absence only, 6; pulsed/continuous US in presence of MN@EP@IP TDD *G. macrophyllus*, 7; Combined modalities NIRL/US only and 8; Combined modalities NIRL/US in presence of MN@EP@IP TDD *G. macrophyllus* on cellular level. [1; untreated EAC implanted group without any treatment, 2; MN@EP@IP TDD *G. macrophyllus* treated group without activation].

The H&E EAC stained sections in all mice study groups illustrating the effects of MN@EP@IP TDD *G. macrophyllus* PDT, SDT, and SPDT on the EAC, are shown in Fig. 4. The histological analysis illustrated that all tumors from the untreated EAC control group were made up entirely of highly cancerous cells and that had 5% necrosis. The EAC tissues histologically exhibited a merely significant changes in the mice treated with MN@EP@IP TDD *G. macrophyllus* alone without activation with regard to the untreated EAC control mice. In addition, all EAC mice treated with laser, ultrasound or combination only demonstrated a considerable areas of necrosis compared with the EAC untreated control group, while the administration of MN@EP@IP TDD *G. macrophyllus* in the laser, ultrasound and combination activated groups demonstrated significantly large foci (80–95%) of necrotic areas with regard to the untreated EAC control mice.

## Discussion

Recent developments in the field of minimally invasive TDD are highlighted in this study. The SC layer complicates the administration of medicinal agents through the skin because it acts as a barrier to entry and slows absorption. Different tactics have been used to improve transdermal permeability for medication delivery of different substances. Physical methods like IP, EP, and MN provide electrical amplification for transdermal biomolecule detection and minimally invasive delivery of amphiphilic therapeutic molecules to the targeted region. IP delivery entails applying small currents to the skin and polarized and neutral molecules moving through it. In the EP technique, tiny pores are created in the skin by applying a 10–1000 voltage for a limited period of time. With a little protrusion in their structure, MNs have a cavity for holding medications^9,15,27–31^. Our study assesses current advancements in the efficiency and design of transdermal systems. A new paradigm for the treatment of local tumors has emerged as a result of the development and adoption of TDD based on the physical enhancement transdermal method (PETDM). Toxic side effects brought on by the first-pass xenobiotic detoxification effect can be avoided if the medicine is given directly to the tumor site through the skin.

The present results showed a significant increased concentration of *G. macrophyllus* that permeated through skin pretreated with EP for 5s, followed by the DC-IP process for 5min at 6, 9 and 12 V with regard to the control group. In addition the experiments results showed that increasing volt decreasing the level of TDD *G. macrophyllus* in EAC bearing mice using three different arrays of MN electrodes (parallel, triangular, and circular). The TDD of *G. macrophyllus* in the EAC bearing mice was maximum possible upon using 6 V parallel MN@ EP@IP. Our study also demonstrated the advantages of MN over transdermal drug delivery techniques due to improved skin penetration that avoids the SC. Additionally, minimally painful trans-epidermal administration systems are paired with penetration augmentation strategies like physical and chemical ways to boost the flow of permeation. These methods, which include IP and EP, are used to physically improve drug administration via the topical route by either altering the SC's structure or by generating pores and micro-channels in the skin. Comparing MN alone to MN with these methods (IP and EP), it is evident that the permeability of medicines across the skin is significantly increased by 5–10 times. This might be accounted for by the fact that the two primary electrical TDD facilitation techniques are EP and IP. Strong electric pulses are momentarily applied to cells during EP, causing the lipid bilayers of the SC to produce aqueous pores, which then allow *G. macrophyllus* to disseminate throughout the skin. It has been shown that delivering high voltage pulses (50-V) for just one second can enhance the transport of a number of medications with a range of molecular weights, from small to large. The process of IP involves pushing charged penetrates into the skin by electrostatic effects and forcing ionic drugs to permeate the skin and enter the body via its potential gradient. This is done by applying physiologically tolerable electrical currents. Since uncharged species can also be given through electroosmosis, unlike other transdermal augmentation techniques, the electrical potential gradient, which acts as a companion to the concentration gradient across the skin, is the main driving force behind how it functions. The main barrier to medications given topically is the SC, which is painlessly bypassed using MN arrays, which are micron-scale, minimally invasive devices. Recent years have seen a lot of research with MN arrays as a way to improve transdermal medicine and vaccine delivery. In contrast to hypodermic needles, the use of MN provided a desirable alternative that has advantages over both those methods. Transdermal patches and MN have also received unprecedented attention for their capacity to increase the skin's permeability to medications^16,17,30,31^.

The present work provides evidence for the oxidants which is being advanced by the EAC. MDA levels were substantially higher in EAC-bearing untreated mice control mice. MDA levels were significantly lower in all MN@EP@IP TDD *G. macrophyllus* treated groups (non activated, activated-laser, active-ultrasound, and activated-combination mice) than in untreated EAC control mice. Furthermore, the antioxidant system was disrupted by severe depletions of the TAC backup as well as the GST, Catalase, GR, and SOD activities. Antioxidant levels were considerably lower in EAC-bearing untreated control mice. All MN@EP@IP TDD *G. macrophyllus* treated groups (non activated, activated-laser, activated-ultrasound, and activated-combination) had significantly higher antioxidant levels than untreated EAC control mice. Previous research has shown that EAC implantation induces a large increase in MDA while decreasing TAC, GST, Catalase, GR, SOD, and activities^32–35^. They discovered that mice implanted with EAC had lower free radical scavenging activity and are more prone to lipid peroxidation than normal healthy animals control mice. As previously indicated, EAC enhances the synthesis of different free radical metabolites, resulting in the overproduction and buildup of ROS and an increase in cell phospholipid polyunsaturated fatty acid bilayer oxidation, consequently raising MDA levels. The fundamental step in the development of oxidative stress and cancer is the depletion of enzymatic and non-enzymatic antioxidants. These enzymatic and non enzymatic antioxidants continuous depletion was illustrate their consumption to detoxify EAC ROS and its metabolites that illustrate the cause for their deficiency. The catalytic function of enzymatic antioxidants may be harmed by the buildup of ROS^36–41^. Our results display how the *G. macrophyllus* capable of scavenge free radical production supports its anti-lipid peroxidative function. In addition also demonstrates the fact of success of *G. macrophyllus* action as SPS and its activation by PDT, SDT and SPDT leading to eradication of EAC the main ROS source so that the antioxidant enzyme activities were higher shifting from cancer state to near normal state.

In our work, EAC implantation caused a significant increase in ALT and AST, indicating liver cell injury in EAC carrying groups. The higher of certain liver enzymes levels in serum are related to those enzymes cellular leakage into the bloodstream, that represents the cell membrane of hepatocyte integrity damage image^42–44^. The findings of this study are consistent with earlier publications in that EAC cancer in mice resulted in a reduction of hepatic function when compared to normal control mice due to the fact that EAC causes organ malfunction and metabolic disruption^32,34–41^. In the current investigation, it was found that *G. macrophyllus* improved ALT and AST levels that is a sign of hepatic protection. Additionally, this supports the *G. macrophyllus* preventive role in preventing hepato-dysfunction brought on by EAC cancer in mice. In this study also, EAC implantation triggered an obvious increment of urea, and creatinine, which indicates kidney injury in EAC bearing groups. Renal dysfunction with increased tubular and glomerular congestion has been documented in EAC-mice as a result of cardiac and hepatic injury, resulting in an increase in renal interstitial pressure on the whole capillary and tubules, which is triggered by an increase in glomerular and tubular congestion. The findings of this study are consistent with earlier publications in that EAC cancer in mice resulted in a reduction of renal function when compared to normal control mice^32,34,41–44^. In the current investigation, it was found that *G. macrophyllus* improved a serum creatinine and urea level that is a sign of kidney protection. Additionally, this supports the *G. macrophyllus* preventive role in preventing renal dysfunction brought on by EAC cancer in mice.

In the present work, the molecular assessment of the expressions of the genes p53, Caspase (3,9), Bax, TNF alpha, Bcl-2, and VEGF as indication for EAC treatment and angiogenesis inhibition respectively revealed a marked positive correlation between the genes' expressions with different treatment modalities in the presence of *G. macrophyllus*, but a negative significant correlation between the genes' expressions and EAC. The expression of the genes p53, caspase 3, 9, TNF alpha and Bax, was significantly higher in the sono-photo-dynamic therapy with (*G. macrophyllus*) mice groups than in the PDT or SDT with (*G. macrophyllus*), followed by IRL or US only without (*G. macrophyllus*), and it was lowest in the EAC untreated mice. In contrast, there was a positive correlation between EAC development and Bcl-2 and VEGF expressions in the untreated EAC mice whereas there was a negative correlation between Bcl-2 and VEGF expressions with different treatment modalities in the presence of (*G. macrophyllus*). Bcl-2 and VEGF gene expressions were lower significantly in mice treated with SPDT therapy with (*G. macrophyllus*) than in mice treated with PDT or SDT with (*G. macrophyllus*) alone, followed by IRL or US alone without (*G. macrophyllus*) alone. Expression was highest in the untreated EAC mice. According to the present work, gene expressions of p53, Caspase (3,9), Bax, TNF alpha, Bcl-2, and VEGF using qRT-PCR was an efficient indicators of cancer efficient treatment, and this finding is similar with other research conducted by other authors^24,33,34^.

Finally, according to the findings, *G. macrophyllus* has the potential to be a photosensitizer and a sonosensitizer for the treatment of in vivo cancer caused by EAC. The *G. macrophyllus* have significant effects on tumour growth inhibition and induction of cell death that may be caused by photo- or sono- chemical activation mechanisms. The use of ultrasound and infrared laser in the presence of *G. macrophyllus* has anticancer effects. The great effectiveness of sonocation followed by light photon irradiation as an anticancer therapy is demonstrated. The findings imply that *G. macrophyllus* has a tremendous potential for usage as a novel sensitizer and as an efficient medication in sono-photodynamic treatment (SPDT).

## Conclusion

The current study yielded substantial findings involving the use of microneedle, iontphrosis, electroporation assisted transdermal drug delivery system (MN@EP@IP@TDDS) in combination with sono-photo-dynamic (SPDT) in presence of *G. macrophyllus* as a sensitizer for treating EAC bearing mice demonstrating promising results for cancer treatment indicated by increasing antioxidants levels, pro-apoptotic genes expression and decreasing oxidative stress level, renal, hepatic biomarkers, anti-apoptotic and angiogenic genes expressions. In addition TDDS has a number of benefits including high bioavailability and minimal systemic toxicity, and become a promising strategy for treating disorders. Furthermore, the combination of MN with IP and EP as PTETs overcomes the TDDS is hindering barrier (SC characteristics, drug and carrier nature, and delivery conditions) as manifested by frozen skin cryo-sections. Moreover, TDD@MN@IP@EP has provided numerous possibilities for antitumor medications in combination with other approaches such as SPDT for successful cancer eradication as illustrated by H&E stained sections, as well as it has paved the way for the deployment of wearable closed-loop customized drug delivery devices in the future.

### Recommendation

The current investigation revealed that microneedle, iontophoresis, electroporation assisted transdermal drug delivery system (MN@EP@IP@TDDS) in combination with sono-photo-dynamic (SPDT) in presence of *G. macrophyllus* as a sensitizer as new promising therapeutic option for cancer treatment that require additional verification. It is strongly advised to carry out more research protocols aimed at safely implementing this modern technique on humans and monitoring changes in various biochemical and/or biophysical parameters.

### Supplementary Information

Supplementary Information.

## Data Availability

All data and materials are included within the manuscript.
