# Supplementary material for: Microneedle combined with iontophoresis and electroporation for assisted transdermal delivery of goniothalamus macrophyllus for enhancement sonophotodynamic activated cancer therapy
Source: Sci Rep. 2024 Apr 4;14:7962. doi: 10.1038/s41598-024-58033-7 (PMC10994924; doi:10.1038/s41598-024-58033-7)
Supplement: Supplementary file 1 — Supplementary Information. [file 41598_2024_58033_MOESM1_ESM.docx]

**Appendix (2)**

**GUIDING PRINCIPLES FOR**

**BIOMEDICAL RESEARCH INVOLVING ANIMALS** https://mri.alexu.edu.eg/images/ArticlesFiles/MRI-Ethics-Code.pdf

1. Methods such as mathematical models, computer simulation and in vitro biological systems should be used whenever appropriate.

2. Animal experiments should be undertaken only after due consideration of their relevance for human or animal health and the advancement of biological knowledge.

3. The animals selected for an experiment should be of an appropriate species and quality, and the minimum number required to obtain scientifically valid results.

4. Investigators and other personnel should never fail to treat animals as sentient, and should regard their proper care and avoidance or minimization of discomfort, distress, or pain as ethical imperatives.

5. Investigators should assume that procedures that would cause pain in human beings cause pain in other vertebrate species, although more needs to be known about the perception of pain in animals.

6. Procedures with animals that may cause more than momentary or minimal pain or distress should be performed with appropriate sedation, analgesia, or anesthesia in accordance with accepted veterinary practice. Surgical or other painful procedures should not be performed on un-anesthetized animals paralyzed by chemical agents.

7. At the end of, or when appropriate during an experiment, animals that would otherwise suffer severe or chronic pain, distress, discomfort, or disablement that cannot be relieved should be painlessly killed.

8. The best possible living conditions should be maintained for animals kept for biomedical purposes. Normally the care of animals should be under supervision of veterinarians having experience in laboratory animal science. In any case, veterinary care should be available as required.

9. It is the responsibility of the director of an institute or department using animals to ensure that investigators and personnel have appropriate qualifications or experience for conducting procedures on animals. Adequate opportunities shall be provided for in-service training, including the proper and humane concern for the animals under their care.
